# Investigation on the passivated Si/Al_2_O_3_ interface fabricated by non-vacuum spatial atomic layer deposition system

**DOI:** 10.1186/s11671-015-0803-9

**Published:** 2015-02-28

**Authors:** Shui-Yang Lien, Chih-Hsiang Yang, Kuei-Ching Wu, Chung-Yuan Kung

**Affiliations:** Department of Materials Science and Engineering, DaYeh University, No. 168, Xuefu Road, Changhua, 515 Taiwan; Department of Electrical Engineering, National Chung Hsing University, 250 State Road, Taichung, 402 Taiwan; Crystalline Silicon R & D Section, Mosel Vitelic Inc, No. 1, Creation Road 1, Hsinchu, 300 Taiwan

**Keywords:** PERC, Non-vacuum spatial atomic layer deposition, Al_2_O_3_/SiN_x_:H stacked rear passivation, Blister, Triple-layer SiO_2_/Al_2_O_3_/SiN_x_, H stacked passivation films

## Abstract

Currently, aluminum oxide stacked with silicon nitride (Al_2_O_3_/SiN_x_:H) is a promising rear passivation material for high-efficiency P-type passivated emitter and rear cell (PERC). It has been indicated that atomic layer deposition system (ALD) is much more suitable to prepare high-quality Al_2_O_3_ films than plasma-enhanced chemical vapor deposition system and other process techniques. In this study, an ultrafast, non-vacuum spatial ALD with the deposition rate of around 10 nm/min, developed by our group, is hired to deposit Al_2_O_3_ films. Upon post-annealing for the Al_2_O_3_ films, the unwanted delamination, regarded as blisters, was found by an optical microscope. This may lead to a worse contact within the Si/Al_2_O_3_ interface, deteriorating the passivation quality. Thin stoichiometric silicon dioxide films prepared on the Si surface prior to Al_2_O_3_ fabrication effectively reduce a considerable amount of blisters. The residual blisters can be further out-gassed when the Al_2_O_3_ films are thinned to 8 nm and annealed above 650°C. Eventually, the entire PERC with the improved triple-layer SiO_2_/Al_2_O_3_/SiN_x_:H stacked passivation film has an obvious gain in open-circuit voltage (*V*_oc_) and short-circuit current (*J*_sc_) because of the increased minority carrier lifetime and internal rear-side reflectance, respectively. The electrical performance of the optimized PERC with the *V*_oc_ of 0.647 V, *J*_sc_ of 38.2 mA/cm^2^, fill factor of 0.776, and the efficiency of 19.18% can be achieved.

## Background

For the past decade years, dielectric films have become promising materials applied in high-efficiency silicon solar cells due to their superior surface passivation effect. An attractive candidate for outstanding Si surface passivation is aluminum oxide (Al_2_O_3_), which can be deposited by physical vapor deposition (PVD) system [1], chemical vapor deposition (CVD) system [2-4], liquid-phase deposition (LPD) technique [5,6], and atomic layer deposition (ALD) system [7-9]. Generally, ALD system is the most suitable choice for the deposition of Al_2_O_3_ owing to some advantages: (i) capable of producing very thin conformal and uniform films, (ii) with large process temperature window, and (iii) able to deposit films on high-aspect-ratio substrates. However, traditional plasma-assist ALD and thermal ALD have an extremely low deposition rate of the order of dozen picometers per second; the industrial application of this technique is chiefly limited to CMOS and DRAM processes [10]. Recently, Al_2_O_3_ films are applied to a noted cell structure so-called passivated emitter and rear cell (PERC) as the passivation layers. The PERC structure which offers the possibility of importantly improved performance over traditional commercial cell design needing only little extra process steps can achieve the efficiency of around 22%. Hence, the PERC structure is going to be the next key product of most solar companies.

For the solar industrials, the deposition of Al_2_O_3_ for PERC is mainly by turn-key plasma-enhanced chemical vapor deposition (PECVD) technique due to its higher production capacity in comparison with ALD system. But the uniformity of the PECVD Al_2_O_3_ films is difficult to control well, making the film thicker at the central region and thinner around the edge of the wafer. A spatial-type ALD with both merits of a high deposition rate and producing films with a high-level uniformity has been proposed to provide a great passivation effect and enhance the production capacity. The precursor TMA (Al(CH_3_)_3_) and reactant water vapor (H_2_O) were used to proceed two half reactions to deposit the Al_2_O_3_ films in spatial ALD. A little amount of hydrogen (H_2_) and H_2_O may remain on the rear-side surface of Si substrate. The blisters which form at the Si/Al_2_O_3_ interface occur under an external load in the presence of a tensile residual stress due to the effusion of H_2_ and H_2_O [11]. The blistering may deteriorate minority carrier lifetime. Several studies have claimed that treating the Al_2_O_3_ films with enough thermal budgets prior to the capping of SiN_x_:H and thinning the thickness Al_2_O_3_ film are two possible ways to conquer this obstacle [12].

In this study, a non-vacuum spatial ALD with a deposition rate of 10 nm/min which is ten times faster than the traditional ALD systems is developed. The fast-growing Al_2_O_3_ films are used as a rear-side passivation layer applied to the P-type PERC structure. In the beginning, the analysis of electrical and structural properties for pure Al_2_O_3_ is characterized. The expected blistering formation is observed through an optical microscope. Two approaches tried to solve the blistering problem as well as to improve the efficiency of PERC. Firstly, a very thin stoichiometric silicon dioxide (SiO_2_) film deposited by inductively coupled plasma chemical vapor deposition (ICPCVD) is inserted into the interface between Al_2_O_3_ and silicon wafer to reduce blisters. In the meantime, SiO_2_ film can further chemically passivate the interface defects. Secondly, reducing the thickness of the Al_2_O_3_ film to lower than 10 nm and increasing the post-annealing temperature to a higher temperature of 650°C to enhance out diffusion of gas. After that, the SiN_x_:H films with abundant hydrogen content prepared by ICPCVD are capped on the Al_2_O_3_ films to enhance the passivation effect by filling dangling bonds. The positive effect of the stacked passivation layer is proven from a quasi-steady-state photo-conductance (QSSPC). The electrical performance for the PERC devices with various rear-side-passivated structures is eventually investigated.

## Methods

Few pieces of 15.6 cm × 15.6 cm shiny etched Cz-Si wafers (P-type, 5 Ω-cm, (100-oriented)) wafers of 200 μm thick were prepared. They were then etched in a chemical polishing solution to remove the saw damages at the edges followed by standard RCA clean. Subsequently, the identical Al_2_O_3_ films were deposited on both sides of the Si wafers to evaluate the passivation effect. Various thicknesses of the Al_2_O_3_ films from 10 to 25 nm were firstly prepared before sending them to the furnace for post-annealing process in N_2_ ambient. The temperature was set from 450°C to 600°C. We prepared other wafers capped with identical thin SiO_2_ films as an interlayer by ICPCVD. The Al_2_O_3_ films were deposited on the top of SiO_2_ films to form stacked structures. Those samples were also annealed in the range from 450°C, 500°C, 550°C, to 600°C, respectively. For a special case, the stacked structures SiO_2_/Al_2_O_3_ with thinner Al_2_O_3_ of about 8 nm were fabricated for comparison. A higher annealing temperature of 650°C was treated on these samples to drive more imbedded gas out diffused. The stacked structures capped with silicon nitride films doped hydrogen (SiN_x_:H) was made by ICPCVD, forming the triple-layer stacked structure of SiO_2_/Al_2_O_3_/SiN_x_. These triple-layer stacked films were then annealed at 450°C for 20 min.

High-resolution scanning electrical microscope (HR-SEM) and optical microscope (OM) were used to observe the thicknesses of the Al_2_O_3_ films and the distribution of blisters, respectively. The wafers were characterized by QSSPC effective lifetime measurement (Sinton Company WCT-120; Sinton Instruments, Boulder, CO, USA). Cross-sectional images of the stacked films were carried out by transmission electron microscope (TEM).

**Figure 1:**
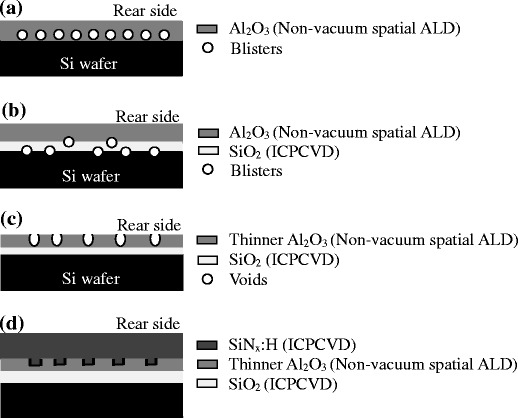
**Introduction for four kinds of structure of rear**-**side passivation films. (a)** Cell A has a pure Al_2_O_3_ film on the rear side of the Si wafer; **(b)** cell B has a thin SiO_2_ film inserted between the Al_2_O_3_ film and Si wafer; **(c)** cell C has the same stacked structure to that of cell B, but thinner Al_2_O_3_ film of 8 nm; and **(d)** cell D has a triple-layer stacked passivation film of SiO_2_/Al_2_O_3_/SiN_x_:H. We introduce the detailed information about the passivation films fabricated in various stages.

After completing the analysis of passivation effect, we started to fabricate the entire PERC devices. For fabrication of emitter, P-type cleaned wafers were thermal diffused by phosphorous atoms in a quartz tube furnace at 850°C. Anti-reflective coatings (ARCs) were deposited by PECVD. Four kinds of the passivation films were prepared on the rear side of wafers as shown in Figure 1. Cell A has a pure Al_2_O_3_ film on the rear side of the Si wafer, having a large number of blisters. Cell B has a thin SiO_2_ film inserted between the Al_2_O_3_ film and Si wafer, having fewer blisters compared to sample A. Note that blisters may probably occur at two interfaces of Al_2_O_3_/SiO_2_ and SiO_2_/Si substrates. According to our experimental results, almost all the blisters are observed to stay at the SiO_2_/Si substrate. This phenomenon can be attributed to the fact that the SiO_2_ thin films fabricated by ICPCVD have lots of pores, allowing H_2_ and H_2_O penetrating into the region between the SiO_2_ layer and the Si substrate after the deposition of Al_2_O_3_. In contrast with cell B, cell C has the same stacked structure, but thinner Al_2_O_3_ film of 8 nm. Post-annealing at 650°C was performed on it as well. Thus, the blisters in cell C were out-gassed, forming some voids to act as defects. The last cell D has a triple-layer stacked passivation film of SiO_2_/Al_2_O_3_/SiN_x_:H as described above. The detailed thickness information for each layer is summarized in Table 1. Laser ablation technique was subsequently used to form the local openings to let the aluminum paste contact with the Si wafer through a co-firing process. I-V characteristics of solar cells were measured using AM1.5G (100 mW/cm^2^) solar simulator.

**Table 1:** **Detailed thickness information of rear**-**side passivation films**

**Cell type**	**SiO**_**2**_ **(nm)**	**Al**_**2**_ **O**_**3**_ **(nm)**	**SiN**_**x**_ **:H (nm)**	**Annealing temperature prior to cap SiN**_**x**_ **:H (°C)**
A	N/A	25	N/A	500
B	3	25	N/A	500
C	3	8	N/A	650
D	3	8	70	650

## Results and discussion

**Figure 2:**
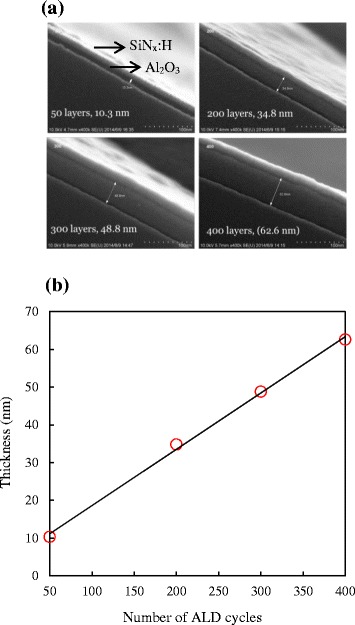
**HR-SEM images for various cycles of deposition and deposition rate of non-vacuum spatial ALD. (a)** HR-SEM images for various cycles of deposition of Al_2_O_3_ films including 50, 200, 300, and 400 cycles and **(b)** deposition rate of non-vacuum spatial ALD. This figure can verify the basic characteristic of deposition rate for a self-developed non-vacuum spatial ALD. The image also shows the uniformity of the Al_2_O_3_ films, revealing its reproducibility.

Figure 2a shows the HR-SEM images for various cycles of deposition of the Al_2_O_3_ films including 50, 100, 300, and 400 cycles. The regime marked by a double-sided arrow is the Al_2_O_3_ film. The aim of capping the SiN_x_ film on the Al_2_O_3_ film is to discriminate each layer to be observed clearly. From this figure, it can be seen that under different deposition cycles, all the Al_2_O_3_ films are uniform without any rough morphology on the surface, revealing the feasibility and reproducibility of this ALD system. Thicknesses of 10.3, 34.8, 48.8, and 62.6 nm correspond to 50, 200, 300, and 400 cycles, respectively. The ALD process allows the deposition of Al_2_O_3_ films with an accurate thickness control is demonstrated in Figure 2b. It is shown that the Al_2_O_3_ film thickness scales near linear with the number of ALD cycles for our non-vacuum spatial ALD. The slope in Figure 2b is defined as growth per cycle (GPC). The GPC here is around 0.16 nm/cycle, 1 s per cycle, so that the deposition rate is around 10 nm/min. Compared to traditional plasma-enhanced ALD and thermal ALD, the deposition rate of 10 nm/min is much faster, displaying its high potential for being used in the industrials.

**Figure 3:**
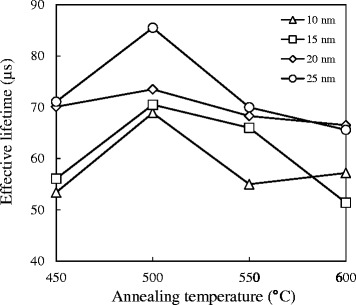
**Minority carrier lifetime for various thicknesses.** Al_2_O_3_ with 10 to 25 nm annealed at 450°C to 600°C in the N_2_ ambient. The effective lifetime was measured by quasi-steady-state photo-conductance (QSSPC) technique to quantify the surface passivation level.

The recombination rate at the Si wafer surface is normally controlled by the excess concentration of minority carriers near the surface. Minimizing the concentration of minority concentration thus reduces the surface recombination rate. Figure 3 shows the minority carrier lifetime for various Al_2_O_3_ film thicknesses with 10 to 25 nm annealed at 450°C to 600°C in the N_2_ ambient. As can be seen in Figure 3, the trends of lifetime for all curves are almost the same, increasing with the increase of temperature firstly and decreasing after at the annealing temperature of 500°C. The decreased lifetime after 500°C can be explained that little bonding structure is broken, releasing few dangling bonds to trap minority carriers. On the other hand, as the thickness of the Al_2_O_3_ film increases, the minority carrier lifetime increases as well. This can be attributed to a lower interface defect density deduced from capacitance voltage measurement for a thicker Al_2_O_3_ film [13]. The peak lifetime 85.5 μs is achieved (the lifetime of bare wafer is about 5 μs), while the thickness of the Al_2_O_3_ film is 25 nm and the annealing temperature is 500°C.

**Figure 4:**
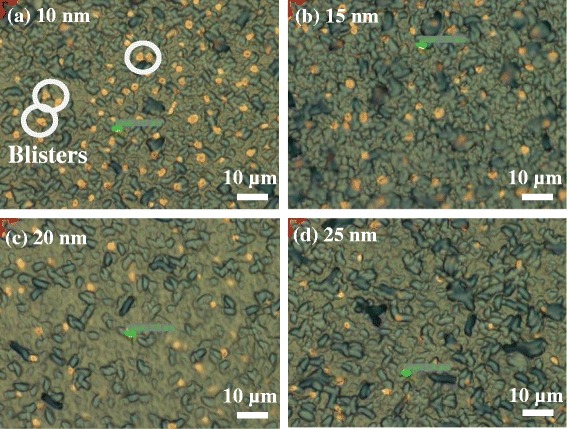
**Optical microscope images for different thicknesses of Al**_ 2_ **O**_ 3_ **film annealed at 500°C. (a)** 10 nm, **(b)** 15 nm, **(c)** 20 nm, **(d)** 25 nm.

In most cases, blister formation caused by the effusion of H_2_O and H_2_ from the silicon bulk may occur upon post-annealing step. Those unwanted blisters are regarded as defects, deteriorating both the chemical effect and field effect of the Al_2_O_3_ films [14]. Figure 4 displays the optical microscope images for different thicknesses of the Al_2_O_3_ film annealed at 500°C: (a) 10 nm, (b) 15 nm, (c) 20 nm, and (d) 25 nm. All the samples have a large number of blisters shown as little spots highlighted by a circle symbol. The diameters of the blister are uniform in the range of 3 ~ 4 μm. With the increase of thickness, the blister density goes lower, resulting in a better passivation effect. The phenomenon can be explained in terms of two aspects: (i) as the Al_2_O_3_ films deposited layer by layer, the weight of entire films becomes heavier, making the blister under the Al_2_O_3_ films dissipate literally; (ii) one Al_2_O_3_ layer forms via the reaction between TMA and H_2_O in sequence on the surface of the silicon wafer. After dozens of cycles, the chemical reaction tends to be stable. The usage of H_2_O raises due to its up and down movement among each porous Al_2_O_3_ layer and chemical reaction with residual TMA at the bottom of the Al_2_O_3_ films. Hence, the amount of the blisters decreases with an increase of the thickness of the Al_2_O_3_. Simultaneously, the distribution of blisters can also be an evidence to account for the lifetime trend in Figure 3.

**Figure 5:**
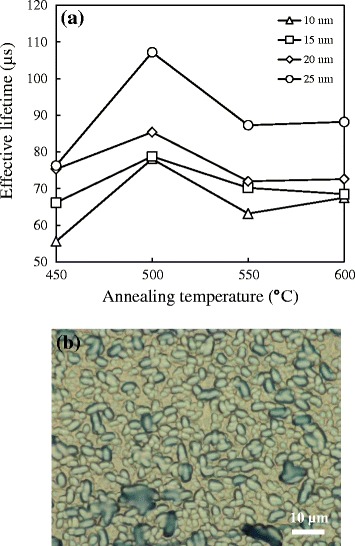
**The blister-blocking effect of SiO**_ 2_ **on silicon. (a)** Minority carrier lifetime for 3 nm SiO_2_ films capped with various Al_2_O_3_ film thicknesses of 10 to 25 nm annealed at 450°C to 600°C in N_2_ ambient and **(b)** optical microscope image for 3 nm-SiO_2_/25 nm-Al_2_O_3_ stacked film annealed at 500°C N_2_ ambient.

The blister-blocking effect of SiO_2_ on silicon can be reflected in Figure 5. Figure 5a shows the minority carrier lifetime for 3 nm of the SiO_2_ films capped with various Al_2_O_3_ films thicknesses of 10 to 25 nm annealed at 450°C to 600°C in the N_2_ ambient. Compared to the trend of Figure 3, it almost maintains unchanged, but the average lifetime of all samples has a little increase. The peak value of 107.2 μs is obtained still when the thickness of the Al_2_O_3_ film is 25 nm, and the annealing temperature is 500°C. The increase of 21.7 μs between two peak lifetime values can be attributed to the enormous reduction of blisters, as shown in Figure 5b. The major reason to support SiO_2_ film to be our option is that the SiO_2_ film has more stoichiometric configuration compared to native oxide (SiO_x_). When the Al_2_O_3_ films deposited directly on the silicon substrate without SiO_2_ films as interlayers, the oxygen atom of reactant H_2_O tends to bond with SiO_x_ to form the stable SiO_2_; thus, the released H_2_ and residual H_2_O may probably become the blisters after post-annealing process. The highly stoichiometric ICPCVD-SiO_2_ films inserted into the interface between the Al_2_O_3_ and silicon wafer effectively prevent the considerable amount of blisters from occurring. In addition, several studies have claimed that SiO_2_ film is a good candidate for chemical passivation to eliminate the dangling bonds on the surface of silicon wafer [15,16]. Also, it can help the Al_2_O_3_ films to rearrange their negative fixed charge distributed near the SiO_2_/Al_2_O_3_ interface [17,18].

**Figure 6:**
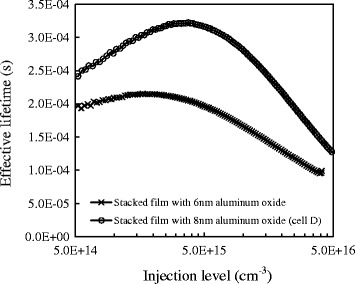
**Injection level dependent minority carrier lifetime for the stacked passivation film.** Si/3 nm-SiO_2_/8 nm-Al_2_O_3_/70 nm-SiN_x_:H film and Si/3 nm-SiO_2_/6 nm-Al_2_O_3_/70 nm-SiN_x_:H film.

For further improvement for the blistering problem, we reduce the thickness of Al_2_O_3_ to lower than 10 nm and increase the annealing temperature to 650°C, giving an enough thermal budget prior to the capping of the SiN_x_:H film. The out-gassing phenomenon can be found. Here, the blister number can further decrease, approaching near blister free. Some voids existing within the Al_2_O_3_ film are caused by the out-gassing effusion. However, the subsequent deposition of SiN_x_:H prepared by ICPCVD would provide abundant hydrogen atoms to fill the dangling bonds via the voids. The post-anneal (450°C for 20 min) performed after the deposition of SiN_x_:H is able to activate the passivation of the triple-layer stacked structure [19]. Figure 6 shows the injection level dependent minority carrier lifetime for the stacked passivation film of Si/3 nm-SiO_2_/8 nm-Al_2_O_3_/70 nm-SiN_x_:H film and of Si/3 nm-SiO_2_/6 nm-Al_2_O_3_/70 nm-SiN_x_:H film. The effective lifetime is calculated from the photoluminescence intensity by the self-consistent calibration method proposed by Trupke et al. [20]. Both the triple-layer stacked films have the same structure except the thickness of the Al_2_O_3_ film. The former one has a higher average lifetime of 315 μs compared to the latter one of 147 μs. Two major factors, negative fixed charge and blisters, are found to influence the lifetime of the Al_2_O_3_ films. Generally, reducing the thickness of the Al_2_O_3_ films to lower than 10 nm and increasing a post-annealing temperature to higher than 650°C can make blisters out-gassed. In this case, both 6- and 8-nm Al_2_O_3_ films are blister free, indicating the lifetime is determined only by negative fixed charge. According to our previous research and some references [21,22], the negative fixed charge may accumulate to enhance the passivation effect as the thickness increases. Hence, the sample with an 8-nm-thick Al_2_O_3_ layer has a higher lifetime, displaying stronger field-effect passivation than the sample with a 6-nm-thick Al_2_O_3_ layer. The optimized lifetime of 315 μs is about three times higher than 107.2 μs of the stacked film without SiN_x_:H. Note that the thickness of the Al_2_O_3_ within the triple-layer stacked film is reduced to lower than 10 nm, decreasing its field-effect passivation. However, according to some investigation of [23-26], they demonstrate that a thin Al_2_O_3_ of about 10 nm is still sufficient for providing an excellent level of surface passivation. Despite the field-effect passivation may become weaker in this case, the chemical passivation from SiN_x_:H dominates the whole performance strongly. For a short summary, hydrogen atom indeed plays a critical role in combing with the Al_2_O_3_ film as the passivation stacks.

**Figure 7:**
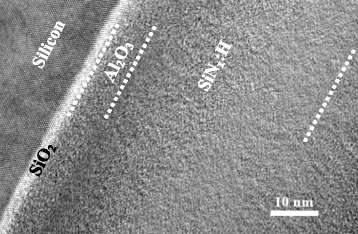
**High-resolution transmission electron microscope (HR-TEM) cross-sectional image of the stacked Si/3 nm-SiO**_ 2_ **/8 nm-Al**_ 2_ **O**_ 3_ **/70 nm-SiN**_ x_ **:H film.**

Figure 7 displays the high-resolution transmission electron microscope (HR-TEM) cross-sectional image of the stacked Si/3 nm-SiO_2_/8 nm-Al_2_O_3_/70 nm-SiN_x_:H film, in which we can see the three interfaces such as Si/SiO_2_, SiO_2_/Al_2_O_3_, and Al_2_O_3_/SiN_x_:H are all flattened without any vacancy or void to deteriorate the passivation effect. The very thin SiO_2_ film with only 3 nm is deposited using the ICPCVD. The accurate control in thickness is based on the deposition rate determined by the past experiments. In the meanwhile, this TEM image confirms that the Al_2_O_3_ film deposited by self-developed non-vacuum spatial ALD is quite uniform.

**Figure 8:**
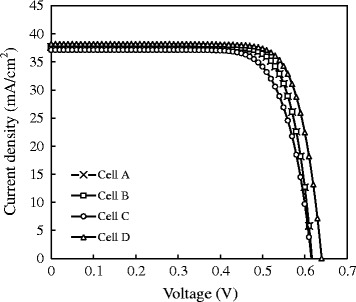
**Reproducible illuminated I-V curves and performance of PERC cells for four PERC structures.**

Figure 8 shows the reproducible illuminated I-V curves and performance of PERC cells for the four kinds of rear-side passivation structure including cell (A) pure 25 nm-Al_2_O_3_ film, (B) 3 nm-SiO_2_/25 nm-Al_2_O_3_ stacked film, (C) 3 nm-SiO_2_/8 nm Al_2_O_3_ film without capping SiN_x_:H treated with an annealing temperature of 650°C, and (D) 3 nm-SiO_2_/8 nm Al_2_O_3_ film/70 nm-SiN_x_:H treated with an annealing temperature of 450°C for 20 min after the capping of SiN_x_:H. All the detailed external parameters are summarized in Table 2. We can find that the electrical performance of cells A and B are almost the same, only with a slight difference in open-circuit voltage (*V*_oc_). To understand the performance *V*_oc_, we investigate the behavior of minority carrier lifetime of the P-type Si wafer. B. Michl et al. have claimed that the excess carrier lifetime substantially affects the *V*_oc_ in multi-crystalline materials [27]. Three equations which can describe the relation between minority carrier lifetime and *V*_oc_ can be expressed by:

**Table 2:** **Photovoltaic performance for PERC cells with various rear**-**side passivation films**

**Cell type**	***V***_**oc**_ **(V)**	***J***_**sc**_ **(mA/cm** ^**2**^ **)**	**FF**	**Efficiency (%)**
A	0.619	37.6	0.78	18.15
B	0.623	37.7	0.782	18.36
C	0.618	37.2	0.748	17.2
D	0.647	38.2	0.776	19.18

1 $$ {V}_{\mathrm{OC}}\kern0.5em =\kern0.5em \frac{kT}{q}\mathrm{In}\left(\frac{J_{\mathrm{SC}}}{J_{\mathrm{os}}\kern0.5em +\kern0.5em {J}_{\mathrm{ob}}}\kern0.5em +\kern0.5em 1\right) $$

2 $$ {J}_{\mathrm{ob}}\kern0.5em =\kern0.5em q\frac{n_i^2}{N_{\mathrm{A}}}\kern0.5em \frac{D_n}{\left({W}_p\kern0.5em -\kern0.5em {X}_p\right)}{\left[1\kern0.5em +\kern0.5em \frac{D_n/\left({W}_p\kern0.5em -\kern0.5em {X}_p\right)}{S_{\mathrm{back}}}\right]}^{-1} $$

3 $$ \frac{1}{\tau_{\mathrm{eff}}}\kern0.5em =\kern0.5em \frac{1}{\tau_{\mathrm{bulk}}}\kern0.5em +\kern0.5em \frac{S_{\mathrm{front}}\kern0.5em +\kern0.5em {S}_{\mathrm{back}}}{W} $$

where *J*_oe_ and *J*_ob_ are the reverse saturation current, respectively. *N*_A_ is doping concentration, *n*_*i*_ is the intrinsic carrier concentration, *S*_back_ is the recombination velocity of back side surface, and *τ*_eff_ and *τ*_bulk_ are effective lifetime and bulk lifetime of devices, respectively. By Equation 3, we can obtain that *S*_back_ may decrease with the increase of effective lifetime. Whereas the smaller *S*_back_ leads to a lower *J*_ob_ expressed in Equation 2. Generally, the value of *J*_ob_ changes its order of magnitude, leading to a huge variation of *V*_oc_. Hence, from Equation 1, an increased *V*_oc_ can be obtained by a reduced *J*_ob_. The deposition of very thin SiO_2_ film in cell B can not only reduce the blister number but also help to rearrange the negative fixed charge near the surface of the Al_2_O_3_ film, thus improving the minority carrier lifetime. According to the explanation above, the higher lifetime of cell B leads to a higher *V*_oc_. As for cell C, it can be seen that all the electrical performances are the worst, especially in fill factor (FF). The factor to influence FF in a solar diode is the contact resistance between metal and semiconductor [28,29]. The blisters in cell C are almost out-gassed, resulting in random distribution of voids. After the laser ablation for the rear contact fabrication, the non-uniform openings can be obtained, forming an unfavorable rear contact. The following high series contact may bring a huge reduction in FF. In comparison with cells A, B, and C, cell D has the apparent improvement in *V*_oc_ and short-circuit current (*J*_sc_). The triple-layer stacked film combines the chemical passivation with field-effect passivation at the same time, leading to a relatively high lifetime of 315 μs. Thus, an optimized *V*_oc_ can be acquired. As to the high *J*_sc_, this can be explained that an optimized rear-side triple-layer stacked passivation also acts as an excellent internal back side reflective coating. By reflecting more long-wavelength light, there is an obvious gain in *J*_sc_ [30]. The final optimal efficiency of the cell D achieves 19.18%.

## Conclusions

In this study, the uniform Al_2_O_3_ films with high reproducibility are fabricated by self-developed non-vacuum spatial ALD system. We report two effective ways to improve the blistering problem upon the annealing after the deposition of Al_2_O_3_, including (i) depositing a thin stoichiometric SiO_2_ film on the surface of the silicon wafer by ICPCVD and (ii) further reducing the thickness of the Al_2_O_3_ film to below 10 nm and provide higher thermal budget to the stacked Si/SiO_2_/Al_2_O_3_ film prior to capping with SiN_x_:H. An obvious improvement on blistering issue can be verified from OM images and minority carrier lifetime measurement. The blisters can be out-gassed when treating the 8-nm thin Al_2_O_3_ film with a 650°C annealing temperature. The subsequent deposition of 70 nm-SiN_x_:H film can not only protect the Al_2_O_3_ film from damage but also provide an effective chemical passivation on the surface of the silicon wafer via the voids. The improved triple-layer stacked Si/3 nm-SiO_2_/8 nm-Al_2_O_3_/70 nm-SiN_x_:H passivation film is successfully applied to PERC device with distinct gains in *V*_oc_ of about 0.03 V and in *J*_sc_ of about 0.6 mA/cm^2^. The final optimal conversion efficiency of 19.18% for the PERC device with the improved stacked passivation film is obtained.

## Abbreviations

ARC: anti-reflective coatings
ALD: atomic layer deposition system
CVD: chemical vapor deposition system
FF: fill factor
GPC: growth per cycle
HR-SEM: high-resolution scanning electron microscopy
HR-TEM: high-resolution transmission electron microscope
ICPCVD: inductively coupled plasma chemical vapor deposition
LPD: liquid-phase deposition technique
*V*_oc_: open-circuit voltage
PERC: passivated emitter and rear cell
PVD: physical vapor deposition system
QSSPC: quasi-steady-state photo-conductance
*J*_sc_: short-circuit current
TEM: transmission electron microscope
